# Vascular ultrasonographic findings in canine patients with clinically diagnosed phlebitis

**DOI:** 10.1111/vru.12805

**Published:** 2019-09-12

**Authors:** Joanna Lodzinska, Hannah Leigh, Magdalena Parys, Tiziana Liuti

**Affiliations:** ^1^ Royal (Dick) School of Veterinary Studies, Easter Bush Campus The University of Edinburgh Roslin UK

**Keywords:** B‐mode, dog, Doppler, thrombophlebitis, thrombosis

## Abstract

Peripheral vein phlebitis (inflammation) is a relatively frequent complication in dogs, however, published information on the ultrasonographic characteristics is currently lacking. This prospective, observational study describes the ultrasound (US) characteristics of normal canine cephalic veins, and veins with clinical phlebitis. Correlations among US findings and between US findings versus time that the intravenous catheter was in place were investigated. Safety of the US procedure was evaluated. Fifty patients were prospectively recruited for the study and 18 met the final inclusion criteria. Each patient underwent daily US examinations and was assessed for multiple criteria (vascular wall appearance, compressibility, spontaneity of flow, color fill, and presence/absence of filling defects, flow contour, direction, non‐pulsatility). Characteristics of normal canine cephalic veins were as follows: smooth and thin wall, complete compressibility, no flow disturbances, no filling defects, smooth flow contours, and unidirectional, non‐pulsatile flow with no turbulence. Characteristics of cephalic veins with clinical phlebitis were as follows: wall thickening (83%), decreased compressibility (55%), filling defects consistent with intraluminal thrombus (55%), vessel wall hyperechogenicity (44%), and abnormal color Doppler flow (39%). Significant correlations were found between Doppler filling defects and compressibility, Doppler filling defects and presumed thrombosis, and compressibility and presumed thrombosis (*P* = .001, *P* = .001, *P* = .000, respectively). No correlation was found between the US findings and time the intravenous catheter was in place. Findings indicated that duplex and compressibility US are feasible and safe methods for characterizing and monitoring cephalic veins in dogs with clinical phlebitis.

**Abbreviations**
US: ultrasound

## 1 INTRODUCTION

The insertion of an intravenous catheter is one of the most commonly performed procedures in hospitalized patients.^1^ Peripheral vein phlebitis, defined as the inflammation of a vein, is a relatively frequent complication and may be chemical, bacterial, or mechanical in origin.^2^, ^3^, ^4^ Phlebitis may cause various degrees of pain, and failure of the intravenous catheter (due to temporary or permanent occlusion) can in turn cause interruption to prescribed therapy. It often compromises future venous access and its advanced stages may be associated with deep venous thrombosis and pulmonary embolism.^5^, ^6^ In humans, ultrasound (US) has led to a refinement in the description of venous anatomy in upper and lower limbs and is widely used to assess superficial vein pathologies, especially phlebitis, thrombosis, and thrombophlebitis.^7^, ^8^, ^9^, ^10^, ^11^, ^12^, ^13^, ^14^ Untreated phlebitis can lead to septicemia; therefore, early detection, decision about intravenous catheter removal, and treatment initiation is crucial.^15^ Multiple grading systems according to the severity of the symptoms have been developed with thrombophlebitis classified as the end stage of phlebitis.^4^ In veterinary practice, phlebitis is most commonly diagnosed based on subjective clinical characteristics such as warmth, pain on injection, swelling, and erythema.^16^, ^17^ The incidence of phlebitis ranges from 3%^16^ to 22%.^17^

Duplex US uses a combination of traditional real‐time grey scale US (B‐mode) and color Doppler sonography, and has become the gold standard in assessing the morphology and hemodynamics of the vascular system in human medicine.^9^ This imaging modality has largely replaced venography in the diagnosis and management of leg vein thrombosis in humans. The procedure can be done at the patient's bed‐side and is widely available.^7^ Several studies have been published documenting its diagnostic accuracy in humans.^8^, ^18^, ^19^, ^20^, ^21^, ^22^ Compressibility US is often combined with duplex US for vascular imaging in humans and involves application of manual compression with enough pressure on the skin to completely obliterate the normal vein lumen.^23^ The appearance of a thrombus has been described as variable, depending on its chronicity. In acute stages, a thrombus may be poorly echogenic and might be more difficult to visualize using B‐mode and Doppler ultrasonography. This is why the standard B‐mode and Doppler US examinations are routinely combined with the compressibility US technique. Multiple reports have been published on vascular US in the abdomen of small animals, however published studies on superficial vascular US are currently lacking.^24^, ^25^

Our hypotheses were that duplex US and compressibility US methods would be feasible and safe for investigation of cephalic veins in canine patients, that US abnormalities would be present in patients with clinically diagnosed phlebitis, and that there would be positive correlations between US findings and time the intravenous catheter was in place. Therefore, the objectives of this study were fourfold: (1) describe the US features of normal canine cephalic veins; (2) describe the US features of cephalic veins in dogs with clinically diagnosed phlebitis; (3) test correlations among US findings and between US findings and time the intravenous catheter was in place; and (4) describe approximate times for performing examinations and any adverse reactions to the scanning procedure.

## 2 MATERIALS AND METHODS

### 2.1 Patient population

All patients recruited for this prospective, observational study were client‐owned dogs admitted to the Hospital for Small Animals, The University of Edinburgh, for daily radiotherapy treatment of various tumors between October 2016 and December 2017. Owner consent was obtained prior to recruitment to the study. All procedures were approved by the University's Veterinary Ethics Research Committee.

The primary inclusion criteria included the following: (1) heart rate, respiratory rate, and pulse quality that were within normal limits and (2) no suggestion of vascular disease on the day of admission. The final inclusion criterion was a clinical diagnosis of cephalic phlebitis by the anesthetist that was based on at least one of the following criteria: erythema, edema, and/or response to injection. Initial exclusion criteria included the following: (1) preexisting known cephalic vein pathology; (2) known peripheral venous catheterization of the cephalic vein less than 7 days prior to the scan, and (3) extremity tumor where the radiotherapy would be delivered in that location. Dogs that did not develop clinical phlebitis were excluded from the analysis. All decisions for study inclusion or exclusion were independently made by a veterinary radiologist in training (J.L.) and were based on medical record entries.

### 2.2 Ultrasound technique

All US studies were performed and interpreted by the same veterinary radiologist in training (J.L.), who was blinded to the results of clinical assessment at the time of scanning and data recording. Each patient underwent ultrasonography of the cephalic vein before any other interventions were carried out on the day of admission to exclude presence of vascular US lesions. Furthermore, to obtain a baseline, all dogs had a repeat US examination after the intravenous catheter was placed later on the same day (day 1) and then underwent daily US examinations of the vein until the day when the intravenous catheter was removed (day 2, day 3, day 4, or day 5). Each patient served as its own control. All examinations were performed on conscious patients in sitting or standing positions. Front limbs were clipped and cephalic veins were scanned from the level of cranioproximal aspect of the radius distally to the venous bifurcation to the accessory cephalic vein before intravenous catheter insertion. After intravenous catheter placement, the scans were performed from the level of cranioproximal aspect of the radius distally as close as possible to the intravenous catheter insertion point. The removal of the intravenous catheter was carried out when clinical signs of phlebitis were evident or on day 5. All ultrasound scans were performed using a commercially available US unit (Esaote MyLab6, Genova, Italy) equipped with the high frequency linear array transducer (10‐18 MHz). One focal zone for the transducer was set at an appropriate level to obtain the best image of the vein under investigation. Gain and dynamic gain control were also set to optimise the image. Vessels were scanned in both sagittal and transverse planes and a consistent image capture protocol was established as follows. In the sagittal plane, the proximal vein was to the left of the screen and distal vein to the right of the screen. In the transverse plane, the lateral aspect of the right limb and medial aspect of the left limb were shown to the left of the screen. B‐mode and Doppler studies were followed by compressibility US examinations. Grey scale images or cine loops were recorded prior to color Doppler examination. Color gain was adjusted to reduce excessive color noise. Pulsed wave Doppler was used to measure blood flow velocity. The analysis was performed keeping the angle between the Doppler beam and the long axis of the vessel at 60°. A beam steering technique was used. The Doppler gate was established between 1 and 2 mm, depending on the diameter of the vessel. The transducer was manipulated to optimize the spectral waveform. Venous compression was applied in the short axis plane along the whole vessel with adequate pressure on the skin to ensure that complete obliteration of the normal vein lumen was achieved.

The veins were assessed by the same observer (J.L.), based on previously published US characteristics of a normal venous vessel for human extremities (Table 1).^23^ All veins were evaluated for these features from Day 1 up to Day 5. Patient compliance and approximate time of examination were also recorded for every procedure.

**Table 1:** Ultrasound characteristics of cephalic veins in dogs without and with clinically diagnosed phlebitis

Ultrasound Characteristic	Normal vein	Veins with clinical phlebitis
Wall assessment	Smooth and thin	Thickened and hyperechoic
Compressibility	Complete	Incomplete
Spontaneity of flow	Present	Present
Consistent color fill	Present	Filling defects present, presumed intraluminal thrombi
Smooth flow contour	Smooth	Smooth
Flow disturbance	Absent	Absent
Unidirectional flow	Present	Present
Non‐pulsatility	Present	Present

Clinical assessments were performed and recorded by multiple anesthesiologists on radiotherapy duty (residents with at least 2 years of clinical experience and board‐certified anesthesiologists). Observers were instructed by an anesthesiologist in training (H.L.) and all data were recorded on a standardized form specifically prepared for the purpose of this study. All decisions were made independently and the anesthesiologists were blinded to the ultrasonographic findings.

### 2.3 Statistical analysis

Statistical analysis was selected and performed by a veterinary radiologist in training (J.L.), as advised by the University's statistician. Data were entered into a spreadsheet for initial analysis (Excel 2016, Microsoft, Inc.) and then into a commercially available statistical analysis program that was used for further evaluation (Minitab 17 Statistical Software 2010, State College, PA: Minitab, Inc.). Descriptive statistics were performed and the Spearman rank‐order correlation coefficient was calculated separately for all defined US findings to investigate their relationships. It was also calculated to investigate the correlation between US findings and the time the intravenous catheter was in place. Statistical significance was defined as *P*‐value < .05.

## 3 RESULTS

### 3.1 Patient population

A total of 50 dogs undergoing daily radiotherapy met initial inclusion criteria for the study. Of those, 18 met the final inclusion criteria. There were 12 females (two entire and 10 neutered) and six males (one entire and five neutered). Median age was 9 years (range: 2‐12 years). Median body weight was 25.0 kg (range: 12.0‐39.6 kg). Represented breeds included Boxer (6/18), Labrador (2/18), and one patient of each of the following breeds: Border Terrier, Cocker Spaniel, Spanish Greyhound, Golden Retriever, Greyhound, Keeshund, Samoyed, Springer Spaniel, Weimaraner, and West Highland White Terrier. Pituitary macroadenoma (4/18) and nasal carcinoma (4/18) were the most common pathologies reported among the sample population. Other tumor types included anal sac adenocarcinoma (3/18), soft tissue sarcoma (3/18), glioma (2/18), and mast cell tumor (2/18). The size of the intravenous catheter was 1″ × 20 gauge in seven patients and 1″ × 22 gauge in 11 patients (Smiths Medical's popular JELCO^®^ I.V. Catheter, Southington, USA). The time the intravenous catheter was in place varied between 3 and 5 days.

### 3.2 Imaging features

Ultrasonographic examinations were successfully performed and well tolerated in all dogs. All three US methods (B‐mode, Doppler, and compressibility) were completed in all the patients and were of diagnostic quality. Visualization of the venous structures was possible in all 18 patients but varied depending on the size of the dog. In smaller patients, the examination took longer and extra care was taken not to compress the lumen during B‐mode and Doppler investigations. The difference in patient positioning did not affect the quality of the examination. In all cases, the US examinations took less than 5 min.

On day 1, before the intravenous catheter was placed, the normal cephalic vein displayed smooth and thin wall that was often difficult to visualize as it blended with the surrounding tissues. Complete compressibility was achieved in all patients along the scanned area. No flow disturbances and no filling defects were observed either on B‐mode or Doppler images. All scanned vessels had smooth flow contours and the flow was unidirectional with no turbulence. In pulsed wave Doppler examinations, the range of velocities of included patients was from 0.02 to 0.09 m/s with a median of 0.06 m/s. Pulsatility was not detected in any of the patients but the signal was generally considered relatively poor and inconsistent.

On the last day of examination (when intravenous catheter was removed), all patients showed at least one ultrasonographic change from baseline. Wall thickening was the most common finding (15/18; 83%). The wall was visible in all patients with clinical phlebitis and the thickness range was 0.5‐1.3 mm with a median of 0.9 mm. Wall thickening was followed by incomplete compressibility (10/18; 55%) and presence of a presumed intraluminal thrombus (10/18; 55%). Eight dogs (8/18; 44%) showed increased vessel wall echogenicity and seven (7/18; 39%) had color Doppler filling defects. Pulsed‐wave Doppler examination was performed in all patients but a consistent reading was achieved only in six dogs. The range of velocities of those patients was from 0.02 to 0.04 m/s with a median of 0.02 m/s. In five of those patients, the reading was lower than on initial examination and in one patient it remained the same (0.02 m/s). No other flow disturbances were identified in this group of patients. The flow was spontaneous and no turbulence or pulsatility changes were identified. Twelve dogs (12/18; 67%) had more than one concurrent ultrasonographic lesion. The US findings from patients with clinical phlebitis are summarized in Table 1.

Statistically significant correlations were found between presence of Doppler flow filling defects and US compressibility (*r* = 0.729, *P*‐value = .001), Doppler flow filling defects and presumed thrombosis (*r* = 0.714, *P*‐value = .001), and US compressibility and presence of presumed thrombus (*r* = 0.970, *P*‐value = 0.000). No significant correlations were found among the remaining ultrasonographic findings and between the US findings and the time the intravenous catheter was in place. All correlation results are presented in Table 2.

**Table 2:** Results of the Spearman rank‐order correlation coefficient analysis for ultrasonographic features and time the intravenous cephalic catheter was in place (n = 18 dogs)

	Wall thickening	Wall echogenicity	Doppler filling defects	Vessel compressibility	Thrombus
Wall echogenicity	*r* = 0.202	−	−	−	−
	*P*‐value = .423				
Doppler filling defects	*r* = 0.103	*r* = 0.675	−	−	−
	*P*‐value = .683	*P*‐value = .002			
Vessel compressibility	*r* = ‐0.402	*r* = 0.284	*r* = 0.729	−	−
	*P*‐value = .098	*P*‐value = .253	*P*‐value = .001		
Thrombus	*r* = ‐0.406	*r* = 0.368	*r* = 0.714	*r* = 0.970	−
	*P*‐value = .095	*P*‐value = .133	*P*‐value = .001	*P*‐value = .000	
Time IVC in place	*r* = ‐0.144	*r* = ‐0.142	*r* = ‐0.096	*r* = 0.143	*r* = 0.246
	*P*‐value = .567	*P*‐value = .573	*P*‐value = .706	*P*‐value = .570	*P*‐value = .325

Abbreviation: IVC, intravenous catheter; *r*, correlation coefficient.

## 4 DISCUSSION

**Figure 1:**
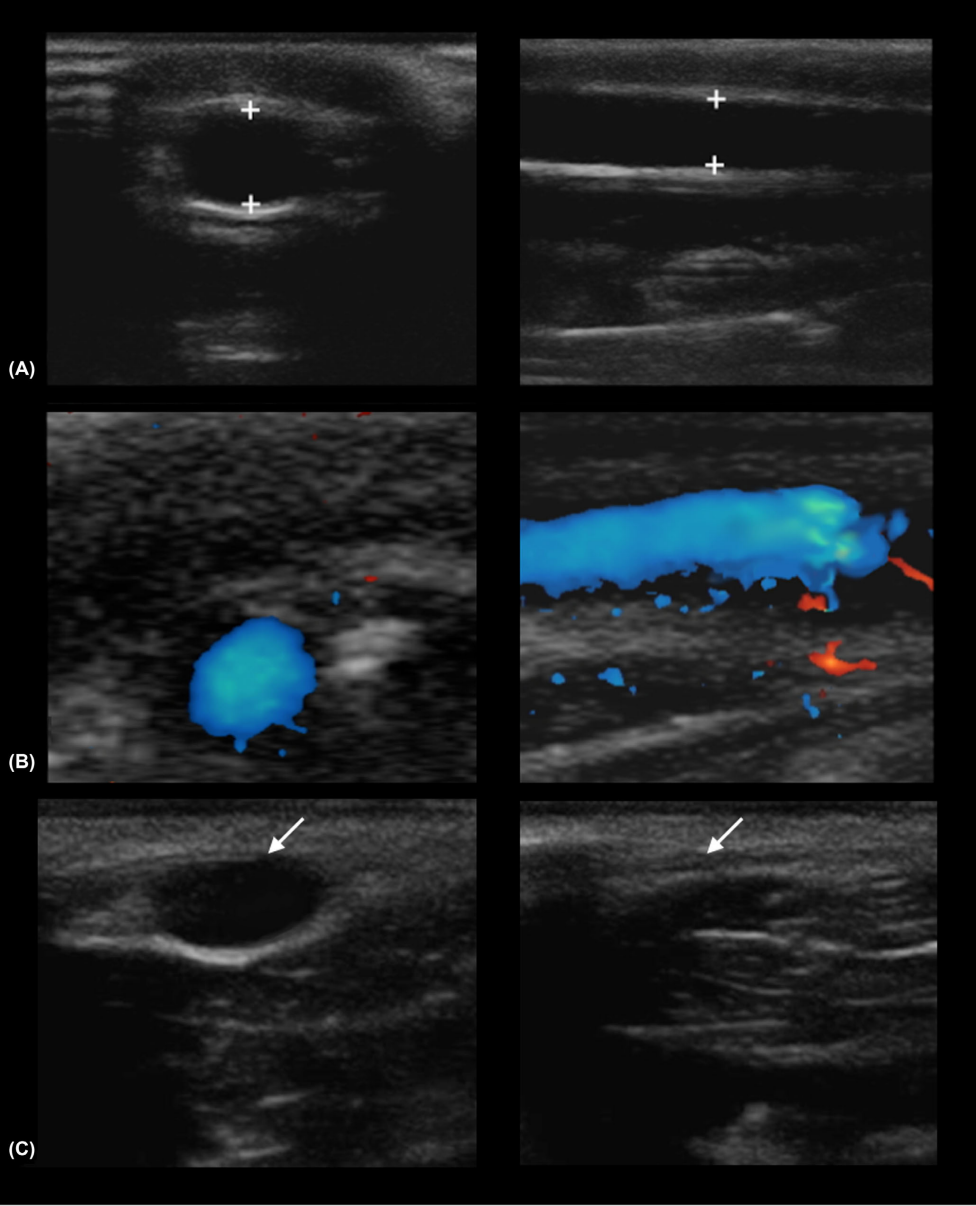
A, Transverse (left image) and sagittal (right image) B mode images of a normal canine cephalic vein (between calipers). B, Normal color Doppler flow. C, The same vessel in transverse plane before (left image) and after (right image) manual pressure has been applied and the vascular lumen has been compressed (arrows). In sagittal images, the proximal vein is to the left of the screen and distal vein to the right of the screen. In transverse plane, the lateral aspect is shown to the left of the screen. Images were acquired with a high frequency linear array transducer (10‐18 MHz)

Based on our review of the literature, the use of duplex US (B‐mode and Doppler) and compressibility US for characterizing normal and pathologic superficial veins of the canine thoracic limb has not been previously published. The ultrasonographic characteristics of normal superficial veins in our sample population of dogs were similar to those reported in humans and are included in Table 1 and Figure 1.^23^ Authors did not identify US abnormalities in the patient cohort examined on day 1. All patients that showed clinical signs consistent with phlebitis also showed US changes.

**Figure 2:**
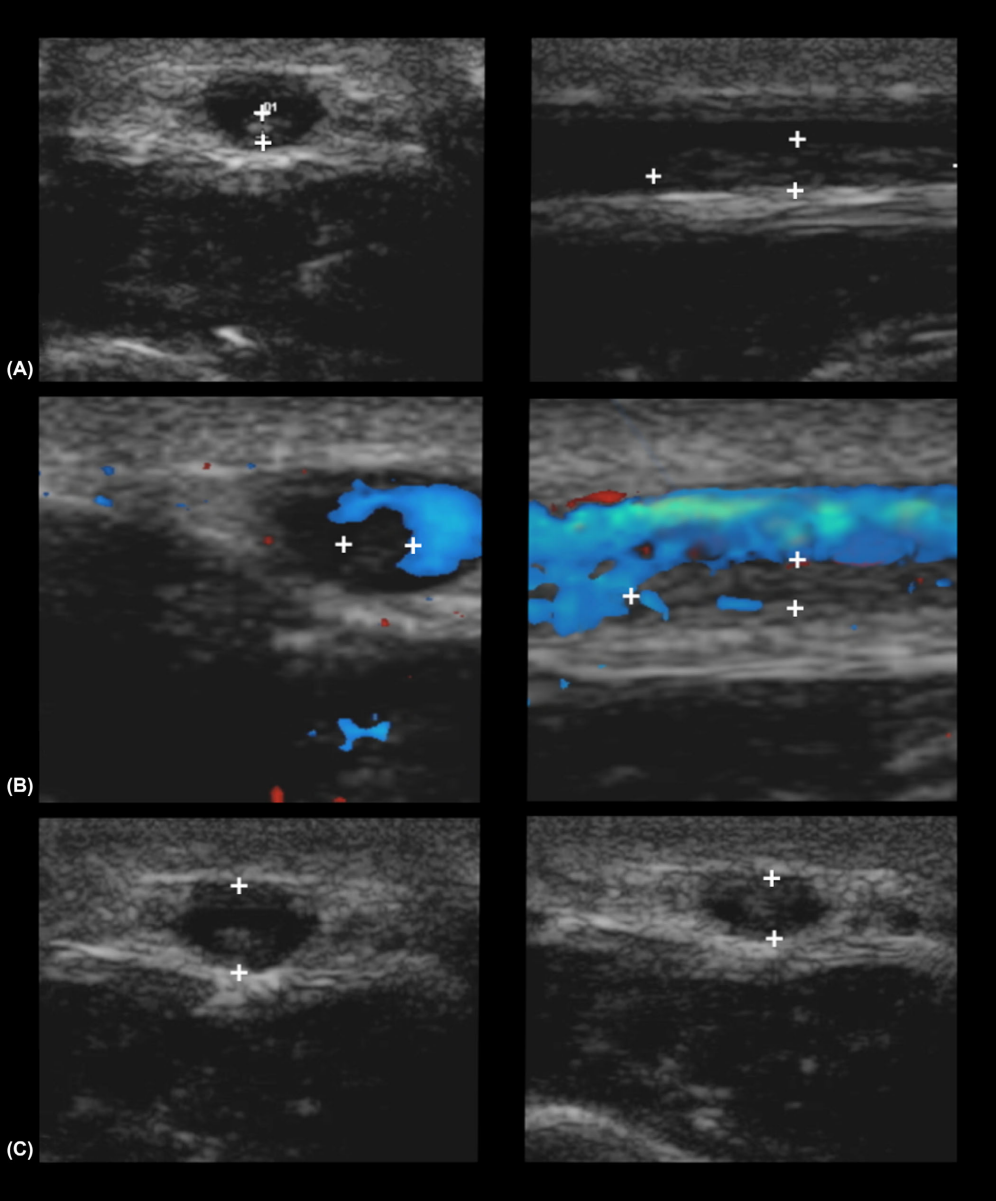
A, Transverse (left image) and sagittal (right image) B mode images of a vein with a presumed intraluminal thrombus (between callipers). B, A thrombotic vessel with color Doppler filling defect. Note the echogenic structure in the far field that represents the thrombus in sagittal plane (between calipers). C, The same vessel in transverse plane before (left image) and after (right image) manual pressure has been applied and the vascular lumen has not been compressed because of the intraluminal structure (between calipers). In sagittal images, the proximal vein is to the left of the screen and distal vein to the right of the screen. In transverse plane, the lateral aspect is shown to the left of the screen. Images were acquired with a high frequency linear array transducer (10‐18 MHz)

Wall thickening was the most common US finding in this sample of dogs. In normal conditions the vascular wall is smooth and thin and often difficult to visualize as it blends with the surrounding tissues.^23^ Any increase in wall thickness should then raise a suspicion of early inflammatory changes. These findings replicate what has been published in human medicine with regards to superficial thrombosis, phlebitis, and thrombophlebitis.^14^, ^23^, ^26^ In veterinary medicine, ultrasonography is also used routinely in horses to diagnose jugular vein thrombophlebitis.^26^, ^27^ The US appearance of abnormal vessels in our patient group, was consistent with that reported in the jugular vein of equine patients.^26^

Presumed intraluminal thrombosis was diagnosed in 10 of our patients, but only seven dogs in our study showed color Doppler filling defects. All 10 patients with presumed intraluminal thrombus had decreased compressibility (Figure 2). One would expect flow disturbances to be present in all the cases of thrombosis, however, our findings were supported by a previous review article in which the disappearance of the color signal was not considered to be a strict requirement for exclusion of thrombosis in humans.^28^ The essential, exclusion criterion for thrombosis was described as complete compressibility of the vein. Lack of compressibility was therefore considered to be diagnostic for thrombosis in humans, as direct visualization of a thrombus could not always be achieved.

Significant correlation was found between US Doppler filling defects and US compressibility, Doppler filling defects and presumed thrombosis, and compressibility and presence of a presumed thrombus. The correlation between these findings was not surprising, as complete compressibility could not be achieved in the presence of an intraluminal structure. Interestingly, no significant correlation was found between the ultrasonographic findings and the time the intravenous catheter was in place. This finding may however change with a larger sample size and serial daily ultrasonographic evaluations. The timing of intravenous catheter removal in cases of phlebitis has only been previously described anecdotally and differs between institutions. Currently in clinical veterinary practice, there is no consensus as to when the intravenous catheter should be removed. The combination of clinical findings and US findings could possibly play an important role in establishing these guidelines in the future.

Time of scanning was short (<5 min) in all cases that makes this method suitable for routine radiological practice as it is very time‐effective. The current study showed that the technique was feasible for a sonographer with moderate ultrasound experience and examinations could be performed in a timely manner. Dogs tolerated the procedure well, since all scans could be performed in standing or seated position. Critically ill dogs may therefore benefit from this scanning protocol, which requires only minimal manual restraint, and short scanning time.

The main limitation of this study was a relatively small sample size. A larger number of dogs would have allowed more accurate description of the findings and determination whether a correlation existed between clinical signs and time the intravenous catheter was in place. The clinically suspected phlebitis was not confirmed histologically; however, this was not possible in our client‐owned patient cohort. In our clinical cases, visualization of the venous structures was possible in all patients but varied depending on the size of the dog. Ultrasound visualization of the cephalic vein in smaller dogs was more challenging compared to larger breeds due to complete collapse of the vessel lumen with probe pressure. Thus, the use of this technique in small breeds of dogs requires more skill and training. This is in accordance with human studies where decreased vessel size prevents US examination.^29^ In this study, the procedure has been performed always by the same operator and this represents a limitation as generalizability of findings for operators with lower or no ultrasound experience was not assessed.

In conclusion, findings supported the use of duplex and compressibility US methods as feasible and safe techniques for evaluation of cephalic veins in dogs with clinical phlebitis. Descriptions of a standardized US protocol and US findings in affected versus unaffected veins were also provided. Future studies are needed to develop consensus guidelines for duration of intravenous catheter placement to help minimize phlebitis in dogs, based on a combination of clinical findings and US findings.

## LIST OF AUTHOR CONTRIBUTIONS

### Category 1

(a) Conception and Design: Lodzinska, Leigh
(b) Acquisition of Data: Lodzinska, Leigh
(c) Analysis and Interpretation of Data: Lodzinska, Leigh, Parys, Liuti

### Category 2

(a) Drafting the Article: Lodzinska
(b) Revising Article for Intellectual Content: Lodzinska, Leigh, Parys, Liuti

### Category 3

(a) Final Approval of the Completed Article: Lodzinska, Leigh, Parys, Liuti
